# The Mediating Role of Resilience in the Association Between Anxiety and Depressive Symptoms Among College Students at a Hispanic-Serving Institution in a U.S.–Mexico Border Region

**DOI:** 10.3390/bs16101802

**Published:** 2026-10-02

**Authors:** Marivic B. Torregosa, Orlando M. Patricio

**Affiliations:** 1 Nursing Department, College of Nursing and Health Sciences, Texas A&M International University, 5201 University Boulevard, Laredo, TX 78041, USA; 2 Natural Sciences and Engineering Department, Laredo College, Laredo, TX 78040, USA; orlando.patricio@laredo.edu

**Keywords:** anxiety, depressive symptoms, resilience, mediation, college students, Hispanic-serving institution, U.S.–Mexico border

## Abstract

Anxiety and depressive symptoms are prevalent concerns among college students, yet less is known about the role of resilience in their association among students in U.S.–Mexico border regions. This cross-sectional study examined whether resilience statistically mediated the association between anxiety and depressive symptoms while controlling for sociodemographic factors and year of data collection. Participants were a convenience sample of 857 college students attending a Hispanic-serving institution in a U.S.–Mexico border region from 2021 to 2025. Anxiety, resilience, and depressive symptoms were measured using the Generalized Anxiety Disorder-7, Brief Resilience Scale, and Patient Health Questionnaire-9, respectively. Mediation analysis was conducted using PROCESS Model 4 with 5000 bootstrap samples. Higher anxiety was associated with lower resilience (*B* = −0.056, *p* < 0.001), and greater resilience was associated with lower depressive symptoms (*B* = −1.379, *p* < 0.001). The indirect association of anxiety with depressive symptoms through resilience was significant (*B* = 0.077, 95% bootstrap CI [0.052, 0.103]). The direct association remained significant (*B* = 0.700, *p* < 0.001), indicating partial statistical mediation. These findings suggest that resilience is an important factor in the association between anxiety and depressive symptoms and may inform comprehensive student mental health initiatives.

## 1. Introduction

Depressive symptoms, anxiety, stress, and poor sleep quality are increasingly prevalent among college students and have become major public health concerns because of their negative effects on academic performance, persistence, and overall well-being (Demenech et al., 2021; Dial et al., 2023; Liu et al., 2023). College students encounter multiple psychosocial stressors, including academic demands, financial hardship, employment responsibilities, and family obligations, all of which may contribute to poor mental health outcomes. Psychological distress among college students has been associated with impaired social functioning, reduced academic success, and increased risk of attrition (Bamber & Kraenzle Schneider, 2016). Although numerous studies have examined the mental health of the general college population, little is known about the role of resilience in promoting psychological well-being, particularly among college students residing in underserved United States (U.S.)–Mexico border regions.

Mental health outcomes among college students are influenced by social determinants of health, including socioeconomic disadvantages, financial insecurity, and environmental stressors. These factors may disproportionately affect college students residing in medically underserved and resource-poor U.S.–Mexico border communities. Border regions tend to experience higher poverty rates, lower median household incomes, reduced healthcare access, and greater economic hardship compared to many non-border regions in the United States (Bastida et al., 2008; Krannich & Massey, 2024). Many students enrolled in colleges in the U.S–Mexico border are first-generation; they frequently experience elevated stress, anxiety, and depressive symptoms related to academic and social challenges (Rockwell & Kimel, 2025). Fear of deportation for themselves or their family members, discrimination, financial insecurity, and limited institutional support negatively affect psychosocial well-being and academic engagement among these students (Nienhusser & Romandia, 2022). First-generation students are also less likely to access mental health services despite being at greater risk for academic attrition (Keefe et al., 2022). Often, these students balance academic responsibilities with employment and family caregiving obligations while simultaneously seeking upward educational mobility (LeBouef & Dworkin, 2021; Presidents’ Alliance on Higher Education and Immigration, 2024). Barriers to financial aid eligibility and fear of deportation despite Deferred Action for Childhood Arrivals (DACA) protections are common concerns for college students in the border regions who do not possess legal immigration documentation (Flores Morales & Garcia, 2021; Gómez & O’Leary, 2019; Suárez-Orozco & López Hernández, 2020), adding to psychological distress. Understanding how resilience may protect against psychological stressors in this college student population is important for developing culturally responsive interventions in higher education, especially at universities in the US–Mexico border region.

### 1.1. Anxiety

Elevated anxiety has been associated with academic difficulties, reduced retention, poorer psychological functioning, and increased use of mental health services (Bamber & Kraenzle Schneider, 2016; Hoeflich et al., 2023). Although depressive symptoms and anxiety are prevalent among college students, many students remain undiagnosed or are hesitant to seek professional mental health care, especially among first-generation students (Keefe et al., 2022; Pérez et al., 2023). College students from U.S.–Mexico border regions often experience increased stress and anxiety associated with acculturative and bicultural stress as they navigate between maintaining cultural traditions and adapting to broader U.S. cultural expectations. Such stressors may contribute to identity conflict, anxiety, depressive symptoms, and maladaptive coping behaviors (Alamilla et al., 2020; Meca et al., 2019; Oshri et al., 2014). Anxiety related to immigration-related issues, fear of potential deportation, and lack of access to resources due to immigration status could contribute to students’ stress and negative psychological outcomes such as depressive symptoms (Flores Morales & Garcia, 2021; Gómez & O’Leary, 2019; Suárez-Orozco & López Hernández, 2020).

### 1.2. Sociodemographic Factors

Sociodemographic and psychosocial factors may put individuals at greater risk for depressive symptoms. Gender differences in mental health outcomes have been consistently reported in the literature. Female college students tend to report higher levels of anxiety, stress, and depressive symptoms than males (Blackdeer et al., 2023; Liu et al., 2023; Pérez et al., 2023). Male college students were found to demonstrate greater resilience following traumatic or stressful experiences (Collen & Onan, 2021; Peng et al., 2022). Age has also been linked with mental health outcomes, with older students generally demonstrating greater emotional regulation, psychological resilience, and perceptions of well-being (Cedillo et al., 2025; Collen & Onan, 2021; Mejia et al., 2022). Financial constraints are a major contributor to poor mental health among college students. Students experiencing food insecurity, housing instability, and financial strain report higher levels of anxiety, depressive symptoms, sleep disturbance, and poorer academic outcomes (Y. Kim & Murphy, 2023; Leung et al., 2021; Liu et al., 2023; Peltz et al., 2021; Uikey & Ingle, 2023). A study conducted at a large 4-year institution revealed that more than half of students experienced at least one form of food, financial, or housing insecurity (Leung et al., 2021). Financial strain and increased work hours have also been associated with sleep disturbance and later depressive symptoms among college students (Peltz et al., 2021). These stressors may be particularly salient among students in resource-limited border communities who often contribute financially to their households while attending college.

### 1.3. Mediating Factor: Resilience

Resilience may lessen the effects of psychosocial stressors on depressive symptoms. Based on resilience theory, individuals with a high sense of resilience consciously or unconsciously choose how to react to disruptive life events and the outcomes from these disruptions. Related to resilience is the concept of reintegration, which involves mind growth, self-knowledge, self-awareness, self-regulation, and increased coping skills derived from experiences of disruptive life events (Richardson, 2017). Reintegration is an individual’s ability to adapt positively to adversity, recover from stressful experiences, and effectively utilize coping resources (Hartley, 2011; Wright et al., 2022). Resilient individuals are more likely to use adaptive coping strategies, positive appraisal, planning, and help-seeking behaviors when faced with stressors (Wright et al., 2022). Prior research has demonstrated that resilience is associated with lower levels of anxiety, stress, and depressive symptoms, and with better academic persistence and psychological well-being among college students (Cassidy et al., 2023; McDermott et al., 2020; Nurius et al., 2023; Ruppamercy et al., 2022). Resilience has also been shown to buffer stress-related factors against depressive symptoms in clinical and nonclinical populations (Assenza et al., 2023; Janitra et al., 2023).

Despite growing evidence linking resilience with better mental health outcomes, important gaps remain in understanding its role among Hispanic college students in underserved U.S.–Mexico border regions. Students in these settings may experience intersecting academic, financial, family, cultural, and immigration-related stressors that contribute to psychological distress. At the same time, cultural and social resources, including family support and cultural values, may contribute to resilience. Existing research supports the relevance of resilience and related protective resources in this population. For example, Dominguez and Brown (2022) found that resilience was associated with better mental health outcomes in a Hispanic community in the U.S.–Mexico border region. Similarly, Morgan Consoli and Llamas (2013) found that Mexican American cultural values, particularly familismo, were associated with resilience among Mexican American college students, while Jimenez et al. (2022) found that family support was associated with lower depressive symptoms and greater college persistence among Mexican-descent college students. However, considerably less is known about whether resilience helps account for the association between anxiety and depressive symptoms among college students attending Hispanic-serving institutions in underserved U.S.–Mexico border communities. This distinction is important because anxiety and depressive symptoms are closely related, yet students experiencing anxiety may differ in their capacity to adapt to and recover from stressful experiences. Examining resilience as a potential statistical mediator may therefore provide a more nuanced understanding of the association between anxiety and depressive symptoms in this population. Such knowledge may also help inform comprehensive student mental health initiatives that address psychological distress while supporting adaptive resources relevant to students in border communities.

**Figure 1:**
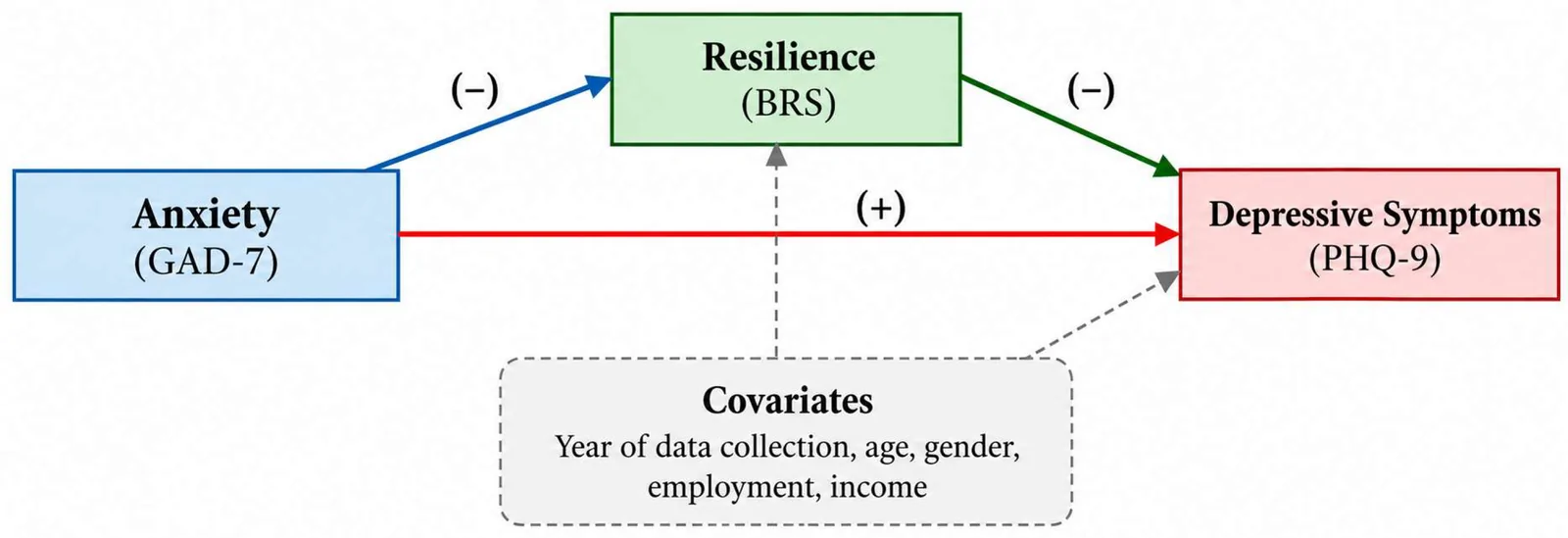
Hypothesized Mediation Model of Resilience in the Association Between Anxiety and Depressive Symptoms. Note: GAD-7 = Generalized Anxiety Disorder-7; BRS = Brief Resilience Scale; PHQ-9 = Patient Health Questionnaire-9. The model hypothesizes that resilience partially mediates the association between anxiety and depressive symptoms while controlling for year of data collection, age, gender, employment, and income.

Therefore, the purpose of this cross-sectional study was to examine whether resilience statistically mediated the association between anxiety and depressive symptoms among college students attending a Hispanic-serving institution in a U.S.–Mexico border region, while controlling for sociodemographic factors and year of data collection. The hypothesized associations among anxiety, resilience, and depressive symptoms, along with the relevant covariates, are depicted in the conceptual model (Figure 1). Based on the extant literature, we proposed the following hypotheses:

**H1.**  *Higher anxiety will be associated with lower resilience and higher depressive symptoms, whereas higher resilience will be associated with lower depressive symptoms.*

**H2.**  *The association between anxiety and depressive symptoms will be partially accounted for by resilience, as evidenced by a significant indirect association between anxiety and depressive symptoms through resilience.*

## 2. Materials and Methods

Using a cross-sectional research design, a survey was administered to a convenience sample of 877 students at a Hispanic-serving institution located along the U.S.–Mexico border. After data screening and application of the age eligibility criterion, the mediation analysis included 857 participants with complete data on the variables included in the final model. Participants were recruited face-to-face by visiting classrooms after coordinating with the course faculty. We also used flyers, social media, and conversations with students during on-campus student events. Although participant recruitment began in 2020, COVID-19-related campus restrictions delayed data collection. Consequently, data were collected from 2021 through 2025. The same recruitment procedures were followed through the years of data collection. The same study questionnaires were delivered via Qualtrics™ across the years of data collection. Texas A&M International University Institutional Review Board #2020-09-04 approval was obtained from 2020 through 2025; informed consent was obtained before data collection. This study was conducted in accordance with the ethical principles outlined in the Declaration of Helsinki. All research procedures involving human participants complied with applicable ethical standards and institutional requirements. Students who participated in the study received certificates documenting volunteer hours for participation. The issuance of certificates also served as a mechanism for tracking participation and minimizing the risk of repeat participation and duplicate responses.

The 7-item Generalized Anxiety Disorder scale (GAD-7) was used to measure anxiety and has demonstrated high internal consistency in prior studies (Kliem et al., 2025; Lutkiewicz et al., 2024; Spitzer et al., 2006; Stefana et al., 2026). The GAD-7 is measured on a 4-point Likert scale (0 = not at all; 1 = several days; 2 = over half the days; 3 = nearly every day). The total score on the GAD-7 was used to measure anxiety, as recommended by the instrument developers (Spitzer et al., 2006). Higher scores indicate greater anxiety symptom severity. For the current study, Cronbach’s α = 0.898.

The mediating variable, resilience, was measured using the Brief Resilience Scale (BRS) (Smith et al., 2008). This instrument has been used in past studies to measure resilience in the general population (Balgiu, 2025; J. Kim et al., 2023) and among college students (Hews-Girard et al., 2026; Karaman et al., 2019; Saha et al., 2025). The BRS is measured on a 5-point Likert scale (1 = strongly disagree; 2 = disagree; 3 = neutral; 4 = agree; 5 = strongly agree). After reverse scoring BRS items 2, 4, and 6, the six items were summed and divided by 6 to obtain the mean BRS score. Thus, scores range from 1 to 5, with higher scores indicating greater resilience or ability to recover from stress (Smith et al., 2008). For the current study, Cronbach’s α = 0.824.

Depressive symptoms, the outcome variable, were measured using the Patient Health Questionnaire-9 (PHQ-9) (Blackwell & McDermott, 2014). The PHQ-9 has been used in previous studies to measure depressive symptoms (Costantini et al., 2021; Levis et al., 2020). The PHQ-9 is measured on a 4-point Likert scale (0 = not at all; 1 = several days; 2 = more than half the days; 3 = nearly every day). Item scores were summed to produce a total score ranging from 0 to 27, with higher scores indicating greater depressive symptom severity. Scores of 0–4, 5–9, 10–14, 15–19, and 20–27 correspond to minimal, mild, moderate, moderately severe, and severe depressive symptoms, respectively. Participants who indicated self-harm and suicidal ideation were referred to the counseling center of the university for further evaluation. For the present analyses, the PHQ-9 total score was treated as a continuous variable. For the current study, Cronbach’s α = 0.870. Type I error rate was set at α = 0.05, and *p* < 0.05 was considered statistically significant.

A mediation analysis using ordinary least squares regression was conducted with Hayes’ PROCESS macro for SPSS Version 29.0.1.0 (Model 4) to examine whether resilience statistically mediated the association between anxiety and depressive symptoms. Sociodemographic characteristics (age, gender, employment status, and income) and year of data collection were included as covariates. The indirect association of anxiety with depressive symptoms through resilience was evaluated using 5000 percentile bootstrap samples and 95% bootstrap confidence intervals. Regression diagnostics indicated heteroskedasticity in the depressive symptoms model; therefore, HC3 heteroskedasticity-consistent standard errors were used for regression coefficients. Diagnostics did not indicate problematic multicollinearity. Anxiety was specified as the predictor (X), resilience as the mediator (M), and depressive symptoms as the outcome (Y).

## 3. Results

### 3.1. Participant Characteristics and Descriptive Statistics

A total of 857 participants were included in the analysis. As shown in Table 1, participants had a mean age of 21.76 years (SD = 2.83; range = 18–34), and the majority were female (83.1%). Approximately 48.0% reported working 1–39 h per week, and 30.7% reported an annual income of $50,000 or greater. Data were collected from 2021 through 2025, with the largest proportion of participants completing the survey in 2023 (36.4%), followed by 2022 (27.1%), 2024 (15.1%), 2021 (13.5%), and 2025 (7.9%). The mean GAD-7 score was 7.82 (SD = 5.77; range = 0–21), the mean BRS score was 3.23 (SD = 0.73; range = 1–5), and the mean PHQ-9 score was 8.50 (SD = 6.05; range = 0–27).

### 3.2. Bivariate Correlations

Pearson correlations were conducted to examine the bivariate associations among anxiety, resilience, and depressive symptoms. As shown in Table 2, anxiety was significantly and negatively correlated with resilience (*r* = −0.456, *p* < 0.001) and significantly and positively correlated with depressive symptoms (*r* = 0.748, *p* < 0.001). Resilience was significantly and negatively correlated with depressive symptoms (*r* = −0.470, *p* < 0.001). These bivariate findings were consistent with the hypothesized directions of the relationships among the primary study variables.

### 3.3. Mediation Analysis

A mediation analysis was conducted using PROCESS Model 4 to examine whether resilience statistically mediated the association between anxiety and depressive symptoms. Year of data collection, age, gender, employment status, and annual income were included as covariates. Year was dummy coded with 2021 as the reference category. The analysis included all 857 participants.

The model predicting resilience was statistically significant, *F*(9, 847) = 26.65, *p* < 0.001, and explained 23.2% of the variance in resilience (*R*^2^ = 0.232). Higher anxiety was significantly associated with lower resilience (*B* = −0.056, HC3 SE = 0.004, *t* = −13.27, *p* < 0.001, 95% CI [−0.064, −0.048]). Among the covariates, gender was associated with resilience (*B* = 0.160, *p* = 0.008), and participants surveyed in 2025 had lower resilience scores than those surveyed in 2021 (*B* = −0.267, *p* = 0.010). Age, employment status, income, and the 2022–2024 year indicators were not statistically significant (Table 3).

The model predicting depressive symptoms was statistically significant, *F*(10, 846) = 114.82, *p* < 0.001, explaining 59.4% of the variance in depressive symptoms (*R*^2^ = 0.594). Anxiety remained significantly and positively associated with depressive symptoms (*B* = 0.700, HC3 *SE* = 0.030, *t* = 23.51, *p* < 0.001, 95% CI [0.642, 0.759]). Resilience was significantly and negatively associated with depressive symptoms (*B* = −1.379, HC3 *SE* = 0.218, *t* = −6.34, *p* < 0.001, 95% CI [−1.806, −0.952]).

Several covariates were also significantly associated with depressive symptoms. Relative to 2021, participants surveyed in 2023 (*B* = −1.366, *p* = 0.001), 2024 (*B* = −1.126, *p* = 0.025), and 2025 (*B* = −1.157, *p* = 0.046) had lower depressive symptom scores. Participants reporting annual income of $50,000 or greater also had lower depressive symptom scores (*B* = −0.736, *p* = 0.009). Year 2022, age, gender, and employment status were not statistically significant.

### 3.4. Total, Direct, and Indirect Associations

The total association between anxiety and depressive symptoms was statistically significant (*B* = 0.778, HC3 *SE* = 0.027, *t* = 28.98, *p* < 0.001, 95% CI [0.725, 0.830]) (Table 3). After resilience was included as the mediator, the direct association remained statistically significant (*B* = 0.700, HC3 *SE* = 0.030, *t* = 23.51, *p* < 0.001, 95% CI [0.642, 0.759]). The indirect association through resilience was statistically significant (*B* = 0.077, Boot*SE* = 0.013, 95% percentile bootstrap CI [0.052, 0.103]). The confidence interval, estimated using 5000 bootstrap samples, did not include zero, supporting a statistically significant indirect association. Because the direct association remained statistically significant, the findings were consistent with partial statistical mediation. The indirect association represented approximately 9.9% of the total association between anxiety and depressive symptoms.

## 4. Discussion

The purpose of this study was to examine whether resilience statistically mediated the association between anxiety and depressive symptoms among college students attending a Hispanic-serving institution in a U.S.–Mexico border region. The findings supported the hypothesized associations: higher anxiety was associated with lower resilience and greater depressive symptoms, while greater resilience was associated with fewer depressive symptoms. In addition, resilience significantly accounted for part of the association between anxiety and depressive symptoms, although the direct association remained significant, indicating partial statistical mediation. These findings extend the literature by demonstrating the relevance of resilience to the association between anxiety and depressive symptoms within an understudied population of college students in a U.S.–Mexico border region. Given the distinctive social, economic, cultural, and educational circumstances that may characterize students in this setting, examining resilience within this context contributes to a more contextually grounded understanding of college student mental health.

The strong positive association between anxiety and depressive symptoms is consistent with the substantial overlap frequently observed between these forms of psychological distress among college students. In the present study, anxiety and depressive symptoms were strongly correlated (*r* = 0.748, *p* < 0.001), and anxiety remained significantly associated with depressive symptoms after adjustment for resilience, year of data collection, age, gender, employment, and income. The total association between anxiety and depressive symptoms was statistically significant (*B* = 0.778, *p* < 0.001), and the direct association remained statistically significant after resilience was incorporated into the model (*B* = 0.700, *p* < 0.001). These findings underscore the close relationship between anxiety and depressive symptoms in this population and suggest the importance of considering co-occurring psychological symptoms when developing approaches to support student mental health.

Resilience was also significantly associated with both anxiety and depressive symptoms. Higher anxiety was associated with lower resilience (*B* = −0.056, *p* < 0.001), even after controlling for the study covariates. In turn, greater resilience was associated with lower depressive symptom scores (*B* = −1.379, *p* < 0.001) after accounting for anxiety and the covariates. The indirect association between anxiety and depressive symptoms through resilience was statistically significant (*B* = 0.077, Boot*SE* = 0.013, 95% percentile bootstrap CI [0.052, 0.103]). Because the bootstrap confidence interval did not include zero, the findings supported a statistically significant indirect association through resilience. Thus, students reporting greater anxiety also tended to report lower resilience, which, in turn, was associated with greater depressive symptoms.

At the same time, the persistence of a statistically significant direct association between anxiety and depressive symptoms (*B* = 0.700, *p* < 0.001) indicates that resilience accounted for only part of this association. The indirect association represented approximately 9.9% of the total association between anxiety and depressive symptoms. This indirect association suggests that resilience may represent one of several factors relevant to the relationship between anxiety and depressive symptoms. Other psychological, social, academic, financial, and environmental factors may also contribute and warrant further investigation. Accordingly, resilience should be considered as one potentially modifiable factor within a broader, multifaceted approach to student mental health rather than as a singular explanation for the association between anxiety and depressive symptoms.

An important contribution of the present analysis was the consideration of year of data collection. Because data were collected from 2021 through 2025, year was included as a covariate to account for potential temporal differences, with 2021 serving as the reference category. Year was not uniformly associated with the study outcomes. In the resilience model, students surveyed in 2025 had significantly lower resilience scores than those surveyed in 2021 (*B* = −0.267, *p* = 0.010), whereas differences for 2022 (*B* = −0.038, *p* = 0.639), 2023 (*B* = −0.122, *p* = 0.109), and 2024 (*B* = −0.170, *p* = 0.066) were not statistically significant. These findings suggest some temporal variation in resilience during the study period but do not indicate a consistent pattern across years.

Year-related differences were also observed for depressive symptoms. Relative to students surveyed in 2021, those surveyed in 2023 (*B* = −1.366, *p* = 0.001), 2024 (*B* = −1.126, *p* = 0.025), and 2025 (*B* = −1.157, *p* = 0.046) had significantly lower depressive symptom scores after adjustment for the other variables in the model. The difference for 2022 was not statistically significant (*B* = −0.261, *p* = 0.540). These findings indicate some temporal variation in depressive symptoms across the study period and support the decision to adjust for year rather than assume that observations collected across multiple years were temporally equivalent. Importantly, the principal associations among anxiety, resilience, and depressive symptoms remained statistically significant after year of data collection was taken into account.

Several sociodemographic findings also warrant consideration. Gender was significantly associated with resilience (*B* = 0.160, *p* = 0.008), whereas age was not statistically significant (*B* = 0.017, *p* = 0.058). Given the coding used in the analysis (female = 0, male = 1), the positive gender coefficient indicates that male participants had higher adjusted resilience scores than female participants. However, neither gender (*B* = −0.382, *p* = 0.293) nor age (*B* = −0.042, *p* = 0.361) was significantly associated with depressive symptoms after anxiety, resilience, and the other covariates were considered. These findings suggest that the observed gender difference in resilience did not correspond to an independent gender difference in depressive symptoms, and age was not independently associated with either resilience or depressive symptoms in the fully adjusted models.

Annual income was independently associated with depressive symptoms. Participants in the higher-income category (≥$50,000) had lower depressive symptom scores than those in the $0–$49,999 category (*B* = −0.736, *p* = 0.009), controlling for the other variables in the model. This association may reflect the relevance of socioeconomic resources to psychological well-being; however, because income was represented using a broad dichotomous measure, the finding should be interpreted cautiously. Employment status was not significantly associated with either resilience (*B* = 0.065, *p* = 0.152) or depressive symptoms (*B* = 0.110, *p* = 0.681). Income was also not significantly associated with resilience (*B* = 0.020, *p* = 0.685). Overall, anxiety and resilience demonstrated more consistent associations with depressive symptoms than most of the measured sociodemographic covariates.

From a practical perspective, the findings suggest that resilience may be relevant to student mental health initiatives, particularly for students experiencing anxiety symptoms. Programs addressing adaptive coping, problem-solving, social support, emotional regulation, and students’ capacity to recover from stressful experiences may warrant consideration alongside established approaches for identifying and addressing anxiety and depressive symptoms. However, the present findings should not be interpreted as evidence that increasing resilience will necessarily reduce depressive symptoms. Because the study was cross-sectional, temporal ordering and causality cannot be established. Longitudinal and intervention studies are needed to determine whether changes in resilience prospectively influence the association between anxiety and depressive symptoms.

The findings may be particularly relevant to Hispanic-serving institutions and universities located in U.S.–Mexico border regions. Students in these settings may encounter intersecting academic, financial, family, cultural, and community demands that influence psychological well-being. Nevertheless, the present study did not directly measure cultural mechanisms such as familism, acculturative experiences, family obligations, perceived discrimination, or culturally specific sources of social support. Consequently, these factors should not be assumed to explain the observed associations. Future research could directly examine culturally relevant protective and risk factors and determine whether they contribute to the relationship among anxiety, resilience, and depressive symptoms. Comparative studies across institutions, geographic regions, socioeconomic circumstances, and student populations would also help determine the extent to which the present findings generalize beyond this specific institutional and border context.

### Strengths and Limitations

Several strengths should be considered. The study included a relatively large sample of 857 students and simultaneously examined anxiety, resilience, and depressive symptoms using established measures. The mediation model also controlled for multiple sociodemographic characteristics, including age, gender, employment, and income, as well as year of data collection. The adjusted depressive symptoms model explained 59.4% of the variance in PHQ-9 scores, indicating that the variables included in the model collectively accounted for a substantial proportion of the variability in depressive symptoms. The use of 5000 bootstrap samples to estimate the confidence interval for the indirect association further strengthened the statistical assessment of mediation.

The study also has several limitations. First, the cross-sectional design prevents conclusions regarding temporal precedence or causality. Although the statistical model specified anxiety as the predictor, resilience as the mediator, and depressive symptoms as the outcome, the data do not establish that anxiety preceded changes in resilience or that resilience subsequently influenced depressive symptoms. Alternative or reciprocal relationships are possible. Accordingly, the mediation findings should be interpreted as evidence of an indirect statistical association rather than a causal pathway.

Second, the use of self-report measures introduces the possibility of response and common-method biases. Although the GAD-7, BRS, and PHQ-9 are established measures, the scores represent self-reported symptoms and resilience rather than clinical diagnoses or objective assessments. Third, participants were recruited from a single Hispanic-serving institution in a U.S.–Mexico border region, which may limit generalizability to students at other institutions or in other geographic and demographic contexts. The use of convenience sampling further limits population-level inference.

Fourth, although year of data collection was included as a covariate and several year differences were identified, adjustment for year cannot fully isolate the effects of the COVID-19 pandemic or other historical and institutional changes occurring between 2021 and 2025. Year represents a broad temporal indicator rather than a direct measure of pandemic exposure or other time-varying experiences. Finally, income and employment were dichotomized for the regression analyses. Such categorization may obscure variation within groups and reduce the ability to detect more nuanced associations.

## 5. Conclusions

This study found that the association between anxiety and depressive symptoms among college students attending a Hispanic-serving institution in a U.S.–Mexico border region was consistent with partial statistical mediation through resilience. Higher anxiety was associated with lower resilience, and lower resilience was associated with greater depressive symptoms; however, a significant direct association between anxiety and depressive symptoms remained after resilience was included in the model. The indirect association through resilience remained statistically significant after adjustment for age, gender, employment, income, and year of data collection. These findings highlight resilience as an important correlate within the anxiety–depressive symptom relationship while recognizing that student psychological well-being is likely influenced by multiple factors. Longitudinal and intervention studies are needed to establish temporal relationships and determine whether interventions that strengthen resilience can contribute to reductions in depressive symptoms among students experiencing anxiety.

## Figures and Tables

**Table 1:** Participant Characteristics and Descriptive Statistics (N = 857).

Variable	*n* (%)	*M*	*SD*	Range
**Year of data collection**				
2021	116 (13.5)	—	—	—
2022	232 (27.1)	—	—	—
2023	312 (36.4)	—	—	—
2024	129 (15.1)	—	—	—
2025	68 (7.9)	—	—	—
**Gender**				
Female	712 (83.1)	—	—	—
Male	145 (16.9)	—	—	—
**Employment status**				
All other employment categories	446 (52.0)	—	—	—
Works 1–39 h/week	411 (48.0)	—	—	—
**Annual income**				
$0–$49,999	594 (69.3)	—	—	—
≥$50,000	263 (30.7)	—	—	—
Age, years	—	21.76	2.83	18–34
Anxiety (GAD-7)	—	7.82	5.77	0–21
Resilience (BRS)	—	3.23	0.73	1–5
Depressive symptoms (PHQ-9)	—	8.50	6.05	0–27

Note. GAD-7 = Generalized Anxiety Disorder-7; BRS = Brief Resilience Scale; PHQ-9 = Patient Health Questionnaire-9. BRS scores represent the mean of the six items after reverse scoring items 2, 4, and 6. Higher GAD-7 and PHQ-9 scores indicate greater anxiety and depressive symptom severity, respectively, whereas higher BRS scores indicate greater resilience.

**Table 2:** Correlations Among Anxiety, Resilience, and Depressive Symptoms (N = 857).

Variable	1	2	3
1. Anxiety (GAD-7)	—		
2. Resilience (BRS)	−0.456 ***	—	
3. Depressive symptoms (PHQ-9)	0.748 ***	−0.470 ***	—

*** *p* < 0.001.

**Table 3:** Regression and Mediation Analysis of the Association Between Anxiety and Depressive Symptoms Through Resilience (N = 857).

Predictor/Effect	*B*	HC3 *SE*	*t*	*p*	95% CI
Model 1: Resilience (BRS) as outcome					
Anxiety (GAD-7)	−0.056	0.004	−13.27	<0.001	[−0.064, −0.048]
Age	0.017	0.009	1.90	0.058	[−0.001, 0.034]
Gender	0.160	0.061	2.64	0.008	[0.041, 0.279]
Works 1–39 h/week	0.065	0.045	1.43	0.152	[−0.024, 0.153]
Annual income ≥ $50,000	0.020	0.048	0.41	0.685	[−0.075, 0.114]
2022	−0.038	0.080	−0.47	0.639	[−0.195, 0.120]
2023	−0.122	0.076	−1.60	0.109	[−0.272, 0.028]
2024	−0.170	0.093	−1.84	0.066	[−0.352, 0.011]
2025	−0.267	0.103	−2.58	0.010	[−0.470, −0.064]
Model 2: Depressive symptoms (PHQ-9) as outcome					
Anxiety (GAD-7)	0.700	0.030	23.51	<0.001	[0.642, 0.759]
Resilience (BRS)	−1.379	0.218	−6.34	<0.001	[−1.806, −0.952]
Age	−0.042	0.046	−0.91	0.361	[−0.133, 0.049]
Gender	−0.382	0.363	−1.05	0.293	[−1.095, 0.331]
Works 1–39 h/week	0.110	0.268	0.41	0.681	[−0.416, 0.637]
Annual income ≥ $50,000	−0.736	0.281	−2.62	0.009	[−1.289, −0.184]
2022	−0.261	0.425	−0.61	0.540	[−1.095, 0.573]
2023	−1.366	0.419	−3.26	0.001	[−2.188, −0.543]
2024	−1.126	0.501	−2.25	0.025	[−2.110, −0.142]
2025	−1.157	0.578	−2.00	0.046	[−2.292, −0.022]
Total, direct, and indirect associations					
Total effect of anxiety on depressive symptoms (c)	0.778	0.027	28.98	<0.001	[0.725, 0.830]
Direct effect of anxiety on depressive symptoms (c′)	0.700	0.030	23.51	<0.001	[0.642, 0.759]
Indirect association through resilience (ab)	0.077	0.013 †	—	—	[0.052, 0.103] †

***Model fit*:** Model 1: *R*^2^ = 0.232, *F*(9, 847) = 26.65, *p* < 0.001. Model 2: *R*^2^ = 0.594, *F*(10, 846) = 114.82, *p* < 0.001. *Note. B* = unstandardized regression coefficient; HC3 *SE* = heteroskedasticity-consistent standard error; CI = confidence interval; GAD-7 = Generalized Anxiety Disorder-7; BRS = Brief Resilience Scale; PHQ-9 = Patient Health Questionnaire-9. For year of data collection, 2021 served as the reference category. Gender was coded 0 = female and 1 = male; employment was coded 0 = all other employment categories and 1 = works 1–39 h/week; annual income was coded 0 = $0–$49,999 and 1 = ≥$50,000. † For the indirect association, *SE* represents the bootstrap standard error, and the 95% CI represents the percentile bootstrap confidence interval based on 5000 bootstrap samples.

## Data Availability

Data and material will be available upon request.
